# Heterogeneity in clinical features and disease severity in ataxia-associated *SYNE1* mutations

**DOI:** 10.1007/s00415-016-8148-6

**Published:** 2016-05-13

**Authors:** Sarah Wiethoff, Joshua Hersheson, Conceicao Bettencourt, Nicholas W. Wood, Henry Houlden

**Affiliations:** 1 Department of Molecular Neuroscience, UCL Institute of Neurology, Queen Square, London, WC1N 3BG UK; 2 Centre for Neurology and Hertie Institute for Clinical Brain Research, Eberhard-Karls-University, Tübingen, Germany; 3 Neurogenetics Laboratory, The National Hospital for Neurology and Neurosurgery, Queen Square, London, WC1N 3BG UK

**Keywords:** Spinocerebellar ataxia, Genotype–phenotype, *SYNE1*, Gene, Mutation, Clinical severity

## Abstract

The autosomal recessive spinocerebellar ataxias are an exciting field of study, with a growing number of causal genes and an expanding phenotypic spectrum. *SYNE1* was originally discovered in 2007 as the causal gene underlying autosomal recessive spinocerebellar ataxia 1, a disease clinically thought to manifest with mainly pure cerebellar ataxia. Since the original report *SYNE1* mutations have also been identified in families with motor neuronopathy and arthrogryposis but few families have been screened as the gene is very large at 146 exons in length. We screened 196 recessive and sporadic ataxia patients for mutations in *SYNE1* using next generation sequencing in order to assess its frequency and extend the clinicogenetic spectrum. We identified four novel truncating mutations spread throughout the *SYNE1* gene from three families living in London that originated from England, Turkey and Sri Lanka. The phenotype was mainly pure cerebellar ataxia in two families, cognitive decline was present in all three families, axonal neuropathy in one family and marked spasticity in the Turkish family, with a range of disease severities. Searching for genotype–phenotype correlations in the *SYNE1* gene, defects located near the 3′ prime end of the gene are more frequently associated with motor neuron or neuromuscular involvement so far. Our data indicate *SYNE1* mutations are not an uncommon cause of recessive ataxia with or without additional clinical features in patients from various ethnicities. The use of next generation sequencing allows the rapid analysis of large genes and will likely reveal more *SYNE1* associated cases and further expand genotype–phenotype correlations.

**Electronic supplementary material:**

The online version of this article (doi:10.1007/s00415-016-8148-6) contains supplementary material, which is available to authorized users.

## Introduction

The recessive spinocerebellar ataxias (ARCAs or SCARs) are a complex group of neurodegenerative conditions with significant genetic and clinical heterogeneity [1]. They are usually characterized by early-onset ataxia with a variable range of other neurological manifestations such as pyramidal signs, endocrine manifestations, epilepsy, cognitive deterioration and peripheral neuropathy [2, 3]. To date, there are over 70 genes that can cause recessive ataxia and this includes 21 SCAR genes [4]. In 2007, *SYNE1* was identified as a cause of pure cerebellar phenotypes. It was termed recessive ataxia of Beauce (SCAR8 or ARCA1, MIM# 610743), since it was originally identified in a number of French-Canadian families originating from the Beauce and Bas-St-Laurent regions of Quebec [5].

Subsequent investigations have identified more than 11 biallelic truncating mutations and one homozygous missense mutation in individuals originating from Canada, France, Brazil and Japan with pure cerebellar ataxia [6–9]. More recently families from Japan and Turkey with homozygous truncating *SYNE1* mutations have been associated with a motor neuron phenotype in addition to cerebellar ataxia [7, 10]. Furthermore, heterozygous missense mutations in *SYNE1* have been identified in dominant muscular dystrophy and two unrelated probands with Emery-Dreifuss muscular dystrophy (MIM# 612998) where only the exons contributing to the muscle specific isoform of *SYNE1* were investigated [11, 12]. Besides, homozygous acceptor splice site mutations two basepairs 5-prime to exon 137 were identified in a consanguineous Palestinian pedigree with myogenic arthrogryposis [13]. In addition defects in *SYNE1* have been shown to be increased in exome sequencing studies of mental retardation and autism [14, 15].

*SYNE1* encodes the spectrin repeat-containing nuclear envelope protein 1, a structural protein expressed in various tissues and believed to link the nucleoskeleton to the inner and outer nuclear membrane, to membranes of cell organelles, to the actin cytoskeleton, and to the sarcomere in muscle [11, 16, 17]. Data from mice suggest a critical role in neurogenesis and neuronal migration for *SYNE1* as an organiser of nucleokinesis in the interplay with other complex proteins such as SYNE2, SUN1 and SUN2 [18]. However, its direct functional role in the human central nervous system, and particularly in the cerebellum, remains understudied.

In this study, we examined an ethnically diverse UK cohort of autosomal recessive families and sporadic cerebellar ataxia patients through a combination of targeted next generation sequencing and exome sequencing, identifying biallelic *SYNE1* mutations in three families from England, Turkey and Sri Lanka with the phenotypes and severities described here.

## Methods

### Patients

All patients were recruited through the Neurogenetics service at the National Hospital for Neurology and Neurosurgery, Queen Square, London and gave informed consent. All patients had a diagnosis of progressive cerebellar ataxia with either known autosomal recessive or presumed sporadic inheritance with early-onset disease. In total 196 patients were screened for *SYNE1* mutations through either exome sequencing (110 patients) or targeted next generation sequencing (86 patients).

### Genetic analysis

DNA was extracted from peripheral leucocytes of all patients in the diagnostic lab, using standard procedures. Additional samples were taken from affected or unaffected relatives to test for mutation segregation where appropriate [19]. Exome sequencing libraries were prepared using Illumina Nextera Rapid Capture Exome Kits following the manufacturer’s recommendation. Libraries were indexed and sequenced on an Illumina HiSeq2500 machine. A custom sequencing panel was designed to amplify the coding exons of *SYNE1* using the Illumina Truseq Custom Amplicon v1.5 Kits. Libraries were prepared in keeping with the standard recommended protocol and then sequenced on an Illumina MiSeq machine. Bioinformatic analysis was the same for both exome sequencing and targeted next generation sequencing: Reads were aligned to the hg19 genome build using Novoalign with variant calling performed using SAMtools and Genome Analysis Toolkit Best Practices (GATK, Broad Institute). Variant annotation was achieved with ANNOVAR and coverage metrics were investigated using a modified in-house Bedtools coverageBed script. All *SYNE1* annotations and mutation locations given below are for the refseq NM_033071 transcript (ENST00000423061).

The final list of called variants in *SYNE1* was filtered according to the following criteria:

(1) nonsynonymous variants present in a homozygous or compound heterozygous state only, (2) Quality >30, (3) depth >10 and (4) minor allele frequency in exome variant server, ExAC and 1000 g <0.005.

Identified variants were confirmed using Sanger sequencing (see Supplementary Table 1 for primers employed) in affected cases and in parents or unaffected siblings where available to confirm segregation or mutation phase for compound heterozygous mutations.

## Results

**Fig. 1:**
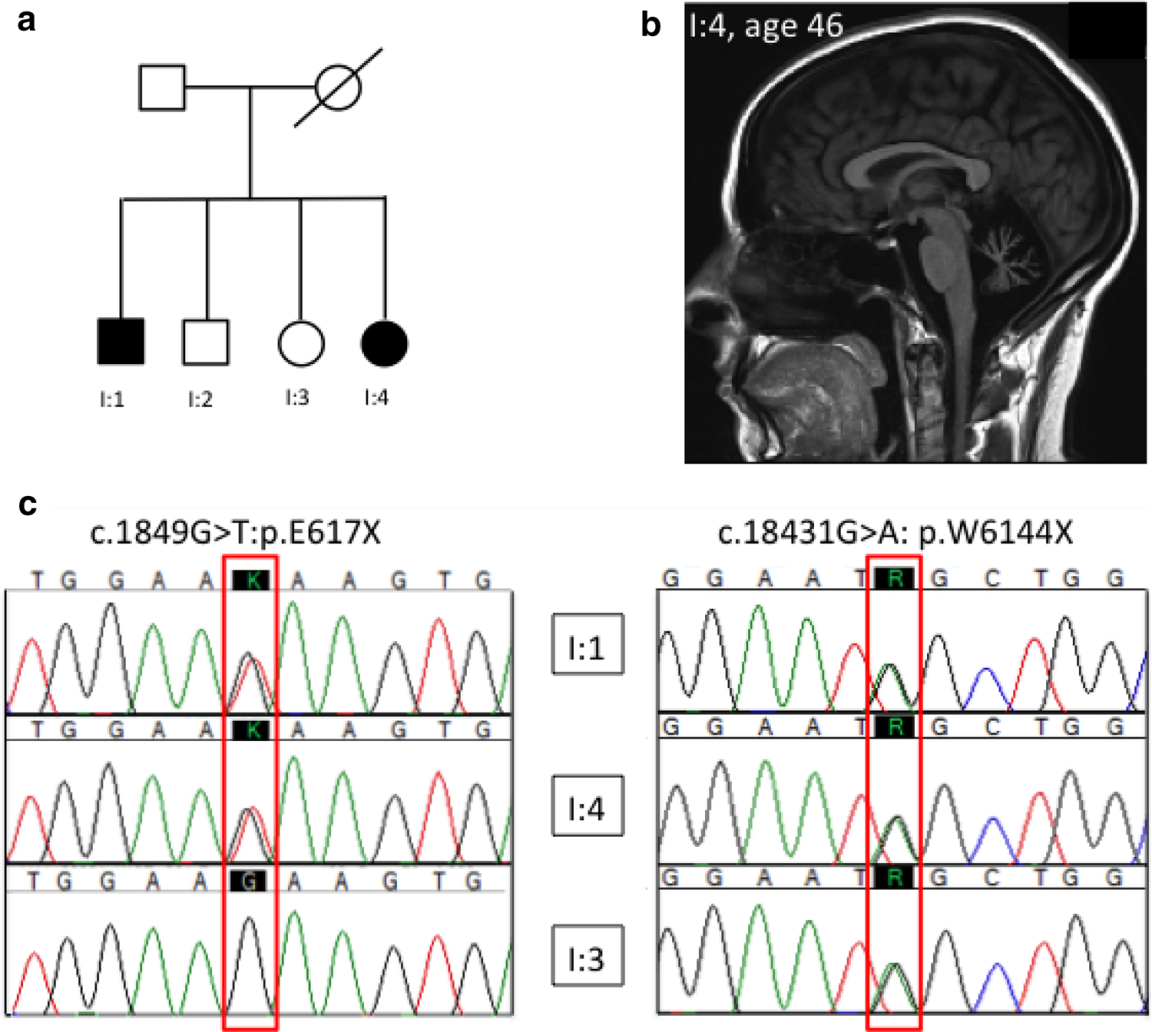
Family tree (**a**), sagittal *T*_1_-weighted MR imaging (**b**) and sequencing chromatograms of two novel *SYNE1* compound heterozygous segregating truncating mutations in family I (c)

Family I—Patient I:1 and I:4 are siblings from a non-consanguineous English family. The two siblings were noted to be poor at sports in school and had poor handwriting. There was, however, no concern about gait or balance until much later in life. Patient I:1 sustained a significant head injury at the age of 21 and was noted to have slightly slurred speech after his accident which was attributed at the time to multiple jaw fractures. There was no perceptible change in gait or coordination until the age of 40 when he noticed difficulty rising from a chair and sustained several falls when walking. He is currently 65 years of age and now has significant gait ataxia and dysarthria. His 49-year-old sister (I:4) did not notice any deterioration in her gait until age 32 after she also was involved in a road traffic accident. She has noticed a slowly progressive deterioration in her gait and speech since this time. Examination of both siblings was very similar with both exhibiting clinical signs of cerebellar ataxia with broken ocular pursuit, cerebellar dysarthria and limb and gait ataxia. Reflexes were normal and there was no clinical and electrophysiologic evidence of neuropathy. Investigations showed both had cerebellar atrophy on MRI (see Fig. 1b for patient I:4).

Cognitive profile of the proband I:4 was as follows: WAIS-III showed verbal IQ of 70 and performance IQ of 67. Verbal subtest scores were impaired (Arithmetic, Digit Span), borderline impaired (Similarities) and low average (Vocabulary). Performance subtest scores were impaired (Block Design) and borderline impaired (Picture Completion, Picture Arrangement). Thus, current performance on the WAIS-III reflects cognitive under-functioning. Overall, memory skills were satisfactory. Thus, performance on a test of verbal recognition memory was good (Recognition Memory Test for Words: 95th percentile). Performance on a test of visual recall memory was good (AMIPB Figure, immediate: 25–50th percentile, and delayed: 50–75th percentile), and performance on a verbal learning task was satisfactory (AMIPB List Learning, A1–A5: 25–50th percentile, A6: 25th percentile). In contrast, performance on a test of visual recognition memory was poor (recognition memory test for faces: 10th percentile). Nominal skills are adequate (graded naming test: 10–25th percentile), and arithmetic skills were impaired (graded difficulty arithmetic test).

Visuospatial skills were weak (VOSP Position Discrimination: <5 % cut-off), and her copy of a complex figure was poor (AMIPB Figure). In contrast, visuoperceptual skills were satisfactory (VOSP incomplete letters: >5 % cut-off). There was evidence of mild executive dysfunction. Thus, phonemic verbal fluency was a little reduced (‘S’: 10), her performance on the Stroop task was impaired (<2nd percentile) and on the Hayling Sentence Completion Task her performance was poor. In contrast, she was able to identify 6/6 categories on the Modified Card Sorting Test, albeit with a little prompting. Speed of information processing was within normal limits (oral symbol digit modalities test). Performance was abnormal on a test of sustained attention (TEA, Elevator Counting: 5/7) and in the borderline impaired range on a test of selective attention (TEA, Elevator Counting with Distraction: 5–10th percentile).

In summary, the cognitive scores of the proband I:4 reflect cognitive under-functioning. On focal cognitive tests, the main findings are weak arithmetic skills, poor visuospatial skills, evidence of executive dysfunction and reduced speed of information processing. Performance on tests of memory and naming were satisfactory, and visuoperceptual skills were intact.

In both affected siblings, two novel compound heterozygous truncating mutations were identified in exon 18 (c.1849G>T:p.E617X) and exon 99 (c.18431G>A: p.W6144X) of *SYNE1*. One of the two unaffected siblings was available for testing and carried only the exon 98 mutation confirming the mutation phase to be trans (see Fig. 1c).

Family II—Patient II:1 is one of 11 siblings from consanguineous parents (1st cousins) of Turkish origin. She was completely well until age 18 years when she developed progressive gait ataxia and dysarthria. At age 23 she was still mobilising without aid but had a high frequency of falls and required adaptations to her home to ensure safety. The patient is now 34 years old. She has 4 affected siblings who all developed symptoms with onset in their late teens and were reported to be very similar to the index case although unfortunately neither clinical notes, nor DNA were available as none of the other family members are resident in the UK and contact has been interrupted. Clinical examination of the index patient showed cognitive difficulties at the bedside but she was not formally assessed. There was broken pursuit eye movements, mild to moderate finger nose ataxia and marked gait ataxia. Reflexes were brisk in the lower limbs with sustained clonus at both ankles and extensor plantar reflexes.

**Fig. 2:**
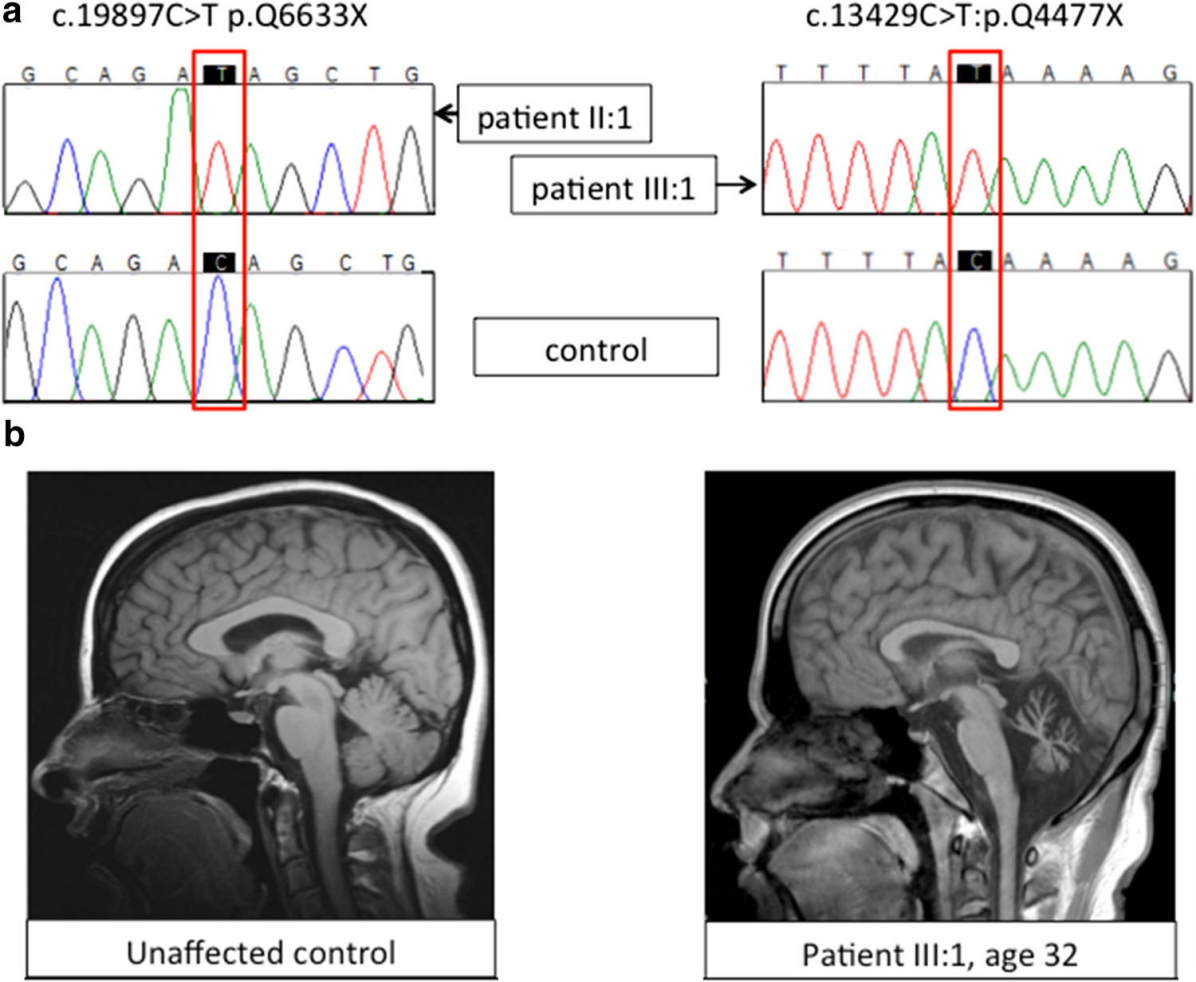
Chromatograms of two novel mutations identified in patient II:1 (*left*) and III:1 (*right*) (**a**) and sagittal *T*_1_-weighted MR imaging of an unaffected control proband, and patient III:1 at age 32 (**b**)

Brain MRI of the index case showed marked cerebellar atrophy. Nerve conduction studies showed no evidence of peripheral neuropathy. A novel homozygous variant in exon 108 of *SYNE1* (c.19897C>T p.Q6633X) was identified in the proband (see Fig. 2a, left panel). DNA for the parents and siblings was not available.

Family III—The proband (III:1) is a 38-year-old Sri Lankan man who originally developed gait and balance problems at 22 years. He is the second of five siblings that are all well and without neurological problems. The patient’s parents were not known to be related. He was fit and active in sports as a child and has despite the obvious gait difficulties remained independent without usage of walking aids till present. Clinical examination showed hypermetric saccades, limb ataxia and extensor plantar reflexes although reflexes were reduced. MRI brain is shown above with cerebellar atrophy (Fig. 2b) and nerve conduction studies showed an axonal neuropathy, other screening blood tests were normal. The examination of this patient is shown on the attached video (see Supplementary Video 1).

Cognitive profile in the proband was as follows: WAIS-III showed a verbal IQ of 76 and performance IQ of 78. Verbal subtest scores were average (Arithmetic) low average (Digit Span), and borderline impaired (Similarities and impaired Vocabulary). Performance subtest scores were low average (Block Design, Picture Arrangement) and borderline impaired (Picture Completion). Performance was normal on a test of verbal memory (short Recognition Memory Test for Words: 75th percentile). In contrast, his scores were poor on two tests of visual memory (Recognition Memory Test for Faces: <10th percentile; AMIPB Figure Recall <5th percentile). Naming skills were satisfactory when considering language and cultural factors. He was able to name 16/30 items on the Oldfield Naming Test. Performance was normal on tests of visual perception and visual spatial functioning (Incomplete Letters: >5 % cut-off, Position Discrimination: >5 % cut-off). Performance was satisfactory on a test of Arithmetic (Graded Difficulty Arithmetic Test: 10–25th percentile). Performance on the simple Stroop test was satisfactory and he passed the Weigl Sorting test. Processing speed is reduced (Symbol Digit Modalities Test <5th percentile). His performance was poor on a test of selective attention (Elevator Counting with Distraction).

In summary, the cognitive scores were mildly impaired on both the verbal and performance scales of the WAIS-III. On a series of focal cognitive tests he presented with a poor performance on some tests of visual memory. In addition, processing speed was reduced and performance was poor on a test of selective attention.

A novel, truncating homozygous variant in *SYNE1*, exon 77 c.13429C>T:p.Q4477X was identified (See Fig. 2a, right panel).

## Discussion

To date, homozygous loss-of-function mutations in *SYNE1* have been reported in French-Canadian, French, Japanese, Turkish and Brazilian individuals [6, 7, 10]. With the first British and Sri Lankan cases detected in this study, we further extend the ethnic diversity underlying *SYNE1* associated cerebellar ataxia. A summary of pheno- and genotype of these and all other recently reported *SYNE1* mutations can be seen in Table 1.

**Table 1:** Reported SYNE1 mutations after the initial discovery in 2007

Family	Case ID	Ethnicity	AAO (years)	Mutation	Clinical features	References
1	I:1	White British	21	p.E617X + p.W6144X	Pure ataxia	NHNN-cohort
2	I:4	White British	32	p.E617X + p.W6144X	Pure ataxia	NHNN-cohort
3	II:1	Turkish	18	hom p.Q6633X	Ataxia + pyramidal tract signs	NHNN-cohort
4	III:1	Sri Lankan	22	hom p.Q4477X	Pure ataxia	NHNN-cohort
5	II:10	French-Canadian	30	p.R125X + p.W6620X	Pure ataxia	[6]
6	III:1	French-Canadian	14	p.R2906X + p.R7084X	Pure ataxia	[6]
7	Case A	Brazilian	na	hom p.Q1300X	Ataxia—no further details	[6]
8	Case B	French	na	hom c.10753-10757delCCAAG/predicted p.R3432Vfs*4	Ataxia—no further details	[6]
9	Patient 1	Japanese	6	hom p.R7486fs7488X (+hom p.G185R)	Ataxia; pyramidal signs, motor neuron disease	[7]
10	Patient 2	Japanese	36	hom p.R3597X	Pure ataxia	[7]
11	Patient 3	Japanese	27	hom p.Y4534fs4539X	Pure ataxia	[7]
12	SYNE1	Turkish	20	hom p.Q7644X (=p.Q7573X for ENST00000423061)	Ataxia; pyramidal tract signs, motor neuron disease	[10]
13	SYNE2	Turkish	24	hom p.R7842X (=p.Q7771X for ENST00000423061)	Ataxia; pyramidal tract signs, motor neuron disease	[10]

Demographic, genetic and clinical summary of SYNE1 mutations reported after the initial discovery paper in 2007

Transcript ENST00000423061/RefSeq protein NP_149062 is used for reference, unless otherwise stated in the table

*AAO* age at onset, *hom* homozygous, *na* not available

**Fig. 3:**
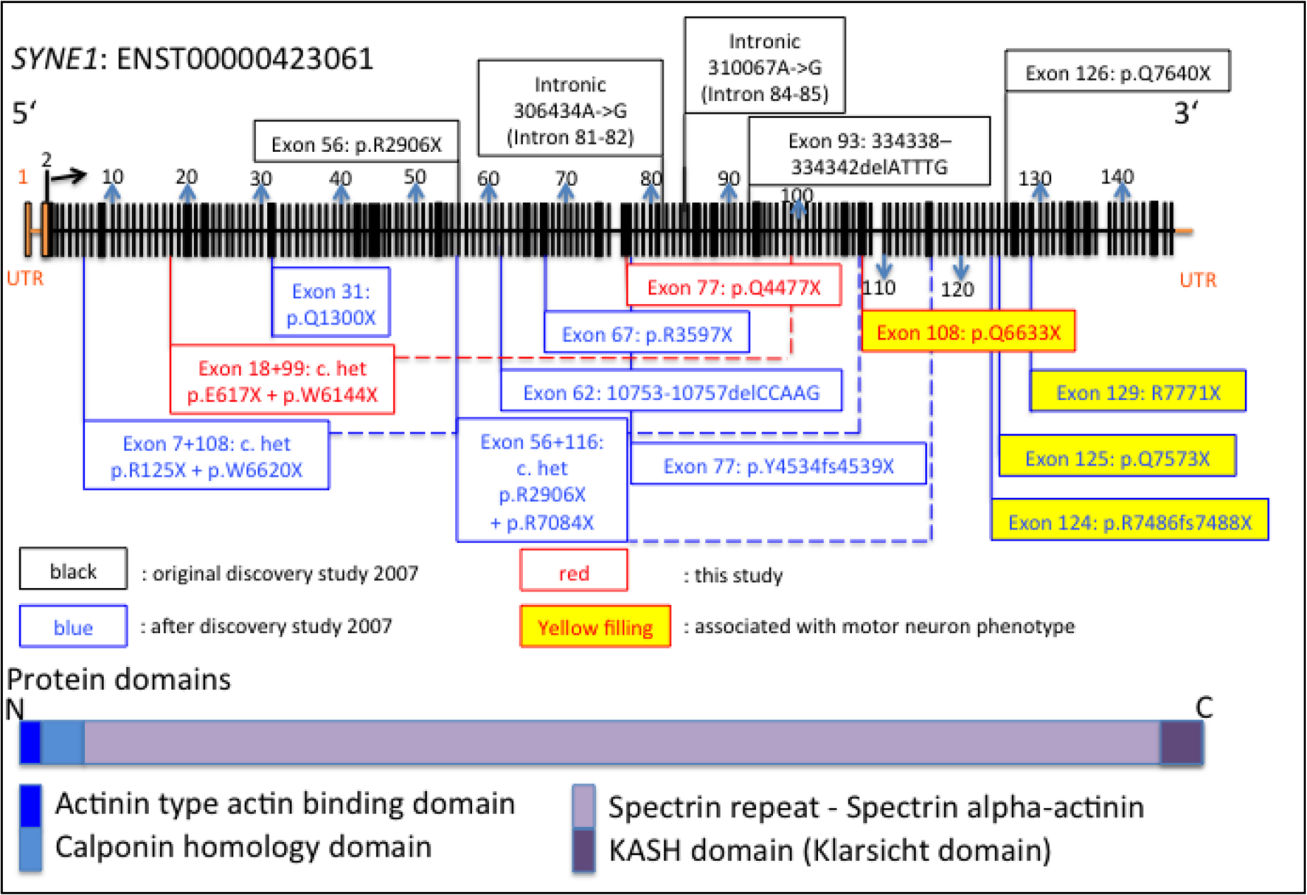
Locations of reported mutations associated with *SYNE1* ataxic phenotypes. Mutations in *black* are from the discovery study, *blue* mutations are mutations reported since and *red* are the mutations identified in our study. *Yellow* *filled boxes* mark mutations associated with motor neuron phenotypes. *Dotted lines* connect the further 3-prime mutation with their partner mutation in compound heterozygous cases. Cave: sizes of *black bars* do not proportionally represent the sizes of the different 146 exons of *SYNE1*

All novel cases reported in our study are associated with truncating mutations likely to result in production of a truncated protein and hence a complete loss or severe reduction of protein function. Clinically, there was a range in the disease age at onset and a possible negative correlation between the position of the stop codon in the reading frame and the severity or age at onset in our cases: e.g., our Turkish case has the most 3-prime mutation of all four; however, this patient presented with the earliest age at onset  (18 years) and additional pyramidal manifestations absent in the other 3 cases. Additionally, looking at cases I:1 and I:4 from our series, phenotypic heterogeneity can be observed within families. Even though based on very low numbers of patients, these observations are mirrored via the other recent reports in the literature (see Table 1; Fig. 3) with *SYNE1* associated cerebellar ataxia being relatively mild and slowly progressive. Our affected cases from families I and III are fairly typical of previously reported cases, although the family from Sri Lanka had an axonal neuropathy. Cognitive decline was evident in all three families, which has been reported in the past in an extensive report on patients from Quebec [20].

The proband from family II, however, exhibited marked lower limb pyramidal signs with hyperreflexia and ankle clonus. Of note, there were no variants in any other ataxia or hereditary spastic paraparesis genes identified in the exome sequence data of this individual. Recently, two other Turkish families have been described with affected members manifesting a spastic ataxia and motor neuron phenotype associated with two novel homozygous *SYNE1* nonsense mutations (R7842X and Q7644X) [10] (also see Fig. 3 for all mutations associated with motor neuron disease as represented by yellow filled boxes).

Interestingly, all recent mutations presenting with significant pyramidal signs were located towards the 3′ prime end of the gene and seemed to be homozygous mutations and not compound heterozygous mutations. However, as with the age at onset and disease severity, this observation is interesting, but based on a very low number of cases. Furthermore, one of the homozygous mutations reported in the initial study in 2007 (p.Q7640X) is located far 3′ prime of the gene as well, but has been reported with pure cerebellar ataxia as part of the initial cohort (see Fig. 3). It is highly likely that further genetic, epigenetic and environmental modifiers are contributing to the phenotypic variability and it is recommended therefore that *SYNE1* mutations be considered also in the aetiology of complex as well as pure recessive ataxia. Further development and establishment of next generation techniques in clinical diagnostics will reduce costs and involved timespans till diagnosis to enable S*YNE1* associated cerebellar ataxia to be more readily identified in the future. However, given the significant size of this gene (146 coding exons, 27,436 base pairs, 8749 amino acids), it is likely to inherently harbour a great deal of genetic variation, and cautious interpretation of identified variants will be needed to correctly infer true pathogenic mutations as opposed to benign polymorphisms.

## Conclusions

Here, we expand the ethnic and genetic diversity of *SYNE1* associated cerebellar ataxia, an important gene to be screened in recessive and sporadic cases. We demonstrate four novel truncating mutations in *SYNE1* in pedigrees from British, Sri Lankan and Turkish origin. We observe a range of inter-family severities, extra-cerebellar and cognitive features and possible genotype–phenotype correlations.

## Electronic supplementary material

Below is the link to the electronic supplementary material.

Supplementary material 1 (MPG 86443 kb)

Supplementary material 2 (DOCX 28 kb)
