# Supplementary material for: Heterogeneity in clinical features and disease severity in ataxia-associated SYNE1 mutations
Source: J Neurol. 2016 May 13;263:1503–10. doi: 10.1007/s00415-016-8148-6 (PMC4971038; doi:10.1007/s00415-016-8148-6)
Supplement: Supplementary file 2 — Supplementary material 2 (DOCX 28 kb) [file 415_2016_8148_MOESM2_ESM.docx]

***Supplementary Table 1:*** Primers for Sanger sequencing of identified mutations in *SYNE1* gene, transcript ID ENST00000423061. *(Abbreviations: x=exon; F=forward; R=reverse; TD=touch down PCR; D=default PCR master mix and sequencing (no DMSO/Solution Q needed))*

| **Primer name** | **Sequence (5’-3’)** | **Amplicon size (bp)** | **Conditions** |
| --- | --- | --- | --- |
| *SYNE1*_x18_F | TGAATGAAACCACCGCTCAG |  |  |
| *SYNE1*_x18_R | GCTACGCTGTAAAAGTCTCTCA | 243 | TD65_55, D |
| *SYNE1*_x98_F | TCCGGTGTGTAGCATGACTT |  |  |
| *SYNE1*_x98_R | TCAGGTTCTTCTCTGGGCAG | 230 | TD65_55, D |
| *SYNE1*_x108_F | GAGAGACTGTCAGGCACGTA |  |  |
| *SYNE1*_x108_R | AATGTGGCGGCTTGTAACAC | 249 | TD65_55, D |
| *SYNE1*_x77_F | AGCCAGCCTCAAGACTTACC |  |  |
| *SYNE1*_x77_R | TGAAGCTCAGAACTCTCATTCA | 297 | TD65_55, D |
